# The Importance of Examining the Teeth

**Published:** 1911-01

**Authors:** 


### The Importance of Examining the Teeth

(*The Hospital*, October. 1910)

THE practitioner is apt to regard the teeth as being the particular province of the dentist. This is a correct attitude, provided that his knowledge is sufficient to enable him to determine when the aid of the dentist should be sought. The latter can only treat cases when called upon to do so, and it frequently happens that irremediable mischief is done before he is consulted. The practitioner is in a position to give the necessary warning. He may, moreover, by judicious advice be the means of preventing disease, and thus obviating pain, inconvenience, and deformity.

### DEVELOPMENT OF THE JAWS.

The development of the jaws is a complex question, but it may be assumed that two very important factors are the preservation of the temporary teeth and the determination of nutrition by the sufficient exercise of the masticatory muscles. Both these factors are regulated by the kind of food eaten by the child. People do not usually go to their dentist for instructions regarding their children's diet, and it therefore behooves the practitioner to be himself in a position to give sound advice on this question. Dr. Sim Wallace has constantly indicated the kind of diet that should be prescribed, and his attitude with regard to the relation that exists between dental caries and diet is summed up in the following quotation: "The cause of the present-day susceptibility to dental caries is that the natural food-stuffs are, to a large extent, deprived of their accompanying fibrous parts and prepared and consumed in a manner which renders them—especially the carbohydrates—liable to lodge and undergo acid fermentation in the mouth, while from the same cause and the induced conditions the acid-producing microörganisms of the mouth lodge and multiply, and augment the rapidity and intensity of the acid fermentation." He pleads for the abolition of "pap" diet after the eruption of the temporary molars and for proper regulation of the foodstuffs, in order that work may be given to structures whose obvious mission is work, and that the teeth may be cleansed by natural means, as opposed to the artificial cleansing brought about by the toothbrush.

### THE MILK TEETH.

It is by no means uncommon in private and hospital practice to hear the condition of temporary teeth regarded by parents and practitioners alike as a matter of no moment. That is an attitude that cannot be too strongly condemned. The association, so frequently found as to be almost constant, of enlarged and septic tonsils and adenoid growths in the naso-pharynx with curious teeth is so striking that one must regard the teeth as the probable primary focus. Certainly the condition of the mouth should receive attention before any operation on the throat is performed. Acute and chronic adenitis may be caused by carious teeth or neglected roots, and the tubercle bacillus may gain entrance by the same channels.

Parents frequently look upon the first permanent molar as a temporary tooth, and this mistake is easily understood when it is remembered that this tooth is the first permanent tooth to erupt, has no predecessor, and takes its place with the temporary teeth. It is a most important tooth, and should be carefully preserved.

### PAIN AND TOOTHACHE.

The frequency with which trigeminal neuralgia is found to originate from some dental lesion is well known. But the cause may not be obvious, and is often only revealed by careful investigation. Local pain may be entirely absent, thus making diagnosis more difficult. The patient, too, is on this account inclined to be skeptical as to the dental origin of the pain. The causative lesions are various, but a decomposing pulp in a tooth free from caries, a chronic pulpitis set up by an amalgam filling, a localised pyorrhea alveolaris, and an apparently efficient crown are among the most obscure. A frequent site of referred pain is the ear, and many an earache has been cured by the extraction of septic roots. Trismus is a symptom not met with very frequently, but its presence should suggest the possible impaction of a lower wisdom tooth, for this is by far the commonest cause of this condition. There is, in connection with swellings about the mouth due to teeth, a popular superstition that nothing should be done till the swelling has gone down. Delay has been productive of much deformity, life has been endangered, and in a few cases death has resulted. Tardy eruption of the temporary teeth is commonly found in rickets.

. . . . . . . . . . . . . . . . . . . . . . .

The mouth should receive proper attention as a preliminary to operations on the stomach, and, indeed, all operations, for the removal of obvious septic foci is only a reasonable procedure. In cases of infective arthritis the condition of the mouth should be carefully investigated, and too much trust must not be placed in antiseptic mouth-washes, for pyorrhea needs rigorous and systematic treatment.

Other conditions in which the teeth need consideration are dyspepsia, chlorotic anemias and pernicious anemia, ulcers of the tongue and consequent epithelioma, affections of the maxillary antrum, and middle-ear disease.

